# Propensity score weighting for addressing under-reporting in mortality surveillance: a proof-of-concept study using the nationally representative mortality data in China

**DOI:** 10.1186/s12963-015-0051-3

**Published:** 2015-07-09

**Authors:** Kang Guo, Peng Yin, Lijun Wang, Yibing Ji, Qingfeng Li, David Bishai, Shiwei Liu, Yunning Liu, Thomas Astell-Burt, Xiaoqi Feng, Jinling You, Jiangmei Liu, Maigeng Zhou

**Affiliations:** National Center for Chronic and Noncommunicable Disease Control and Prevention, Chinese Center for Disease Control and Prevention, 27 Nanwei Road, Xicheng District, 100050 Beijing, China; Department of Medical Statistics, West China School of Public Health, Sichuan University, Chengdu, China; School of Public Health, Johns Hopkins University, Baltimore, USA; School of Science and Health, University of Western Sydney, Sydney, Australia; School of Geography and Geosciences, University of St Andrews, St Andrews, UK; Centre for Health Research, School of Medicine, University of Western Sydney, Sydney, Australia

**Keywords:** Mortality, Surveillance, Under-reporting, Propensity scores

## Abstract

**Background:**

National mortality data are obtained routinely by the Disease Surveillance Points system (DSPs) in China and under-reporting is a big challenge in mortality surveillance.

**Methods:**

We carried out an under-reporting field survey in all 161 DSP sites to collect death cases during 2009–2011, using a multi-stage stratified sampling. To identify under-reporting, death data were matched between field survey system and the routine online surveillance system by an automatic computer checking followed by a thorough manual verification. We used a propensity score (PS) weighting method based on a logistic regression to calculate the under-reporting rate in different groups classified by age, gender, urban/rural residency, geographic locations and other mortality related variables. For comparison purposes, we also calculated the under-reporting rate by using capture-mark-recapture (CMR) method.

**Results:**

There were no significant differences between the field survey system and routine online surveillance system in terms of age group, causes of death, highest level of diagnosis and diagnostic basis. The overall under-reporting rate in the DSPs was 12.9 % (95%CI 11.2 %, 14.6 %) based on PS. The under-reporting rate was higher in the west (18.8 %, 95%CI 16.5 %, 21.0 %) than the east (10.1 %, 95%CI 8.6 %, 11.3 %) and central regions (11.2 %, 95%CI 9.6 %, 12.7 %). Among all age groups, the under-reporting rate was highest in the 0–5 year group (23.7 %, 95%CI 16.1 %, 35.5 %) and lowest in the 65 years and above group (12.4 %, 95%CI 10.9 %, 13.6 %). The under-reporting rates in each group by PS were similar to the results calculated by the CMR methods.

**Conclusions:**

The mortality data from the DSP system in China needs to be adjusted. Compared to the commonly used CMR method in the estimation of under-reporting rate, the results of propensity score weighting method are similar but more flexible when calculating the under-reporting rates in different groups. Propensity score weighting is suitable to adjust DSP data and can be used to address under-reporting in mortality surveillance in China.

## Introduction

Cause of death data are fundamental to developing effective public health policies [1]. Achieving complete vital registration remains difficult for a middle-income country like China with 1.3 billion population and limited resources. As an interim approach, China developed the Disease Surveillance Points System (DSPs) to obtain national mortality data based on multi-stage stratified clustering sampling method [2].

The DSP method is not without limitations and one key challenge is under-reporting of mortality counts. To ensure data integrity, it is necessary to measure the degree of under-reporting. The capture-mark-recapture method (CMR) was used in previous under-reporting surveys in China to correct for under-reporting rate using household survey as a gold standard [3–5]. Using CMR to estimate the under-reporting rate is relatively straightforward and practical, but the assumptions applied for CMR in under-reporting surveys cannot always be met and results could produce biased estimates if covariance distribution between groups is uneven. Therefore, potential alternatives to CMR need to be identified and tested to derive more reliable under-reporting rates for correction of mortality rates.

The purpose of this paper was to introduce a propensity score (PS) weighting method with a logistic regression to offer an alternative correction for under-reporting. This paper used data from an under-reporting survey during the period 2009–2011 to assess the degree of under-reporting of death causes surveillance in the DSP System. In this paper we compared and cross validated the CMR and propensity score weighting methods as options to correct for under-reporting.

## Methods

### The China Disease Surveillance Points System

The DSP was initiated in 1978 and adjusted three times in 1990, 2005 and 2010 on the basis of economic development, geographic location, Gross Domestic Product (GDP), proportion of non-agricultural population and the total population of the country to ensure representativeness. After adjustment in 2010, the DSP system included 64 urban and 97 rural surveillance sites in all 31 provinces (autonomous regions and municipalities) covering seven percent of the total population in China. The information provided by the system can be used to estimate causes of death among the national population and the detailed description of DSPs has been published elsewhere [2, 6]. In brief, all deaths were reported in the monitoring stations in the hospitals, community health centers and village clinics in each DSP based on death certificates. Data on demographics, date of death, place of death, cause of death, and main symptoms and signs (for verbal autopsy), etc., were collected. The 161 DSP-level and 31 provincial-level Centers for Disease Control and Prevention (CDC) were responsible for data quality through regular checking, supervision, feedback and verification. Starting in 2008, all the deaths in DSPs were reported through an online death causes monitoring system.

### Survey of the under-reporting death cases in China

To address the under-reporting, periodic evaluations for completeness of registration were conducted once every three years in DSPs. Two under-reporting field surveys have been carried out during the period 2006–2008 and 2009–2011 respectively. The survey in 2006–2008 showed that the national total crude rate of under-reporting was 16.7 % and the weighted rate was 17.4 %; the under-reporting rate for children aged 5 years and below (35.0 %) was much higher than that for people above age 5 (16.9 %) [7].

#### Field survey design

An under-reporting survey was conducted in all 161 DSPs from July to October in 2012. Within each DSP, three townships (in rural areas) or streets (in urban areas) whose crude death rate (CDR) was close to that DSP’s average CDR were first selected as candidate fields for the under-reporting survey. One township/street was finally chosen as the field site if its economic level was similar to the DSP’s average and the population size was in the middle level among all the townships/streets in the DSP. All the residents in the selected township/street were included as the survey population. Deaths occurring from January 1^st^, 2009 to December 31^st^, 2011 in the families were investigated using interviews with the surviving household residents. The information of death population collected in the field survey included demographics, death-related information such as causes of death, highest level of hospital where illness was diagnosed, and diagnostic basis.

#### Data collection

A list of decedents from the focal time period was created for each resident group (the smallest administrative unit) within all villages and communities in the selected townships or streets by recall of the resident group leaders. The initial list was checked and complemented by data from public security departments, civil affairs departments, family planning departments, and maternal and child health departments. Using the final list of deaths, the interviewers in each village or community surveyed each family which experienced a death to verify and revise relevant information on the death records.

#### Identification of missed deaths

Death records between the field survey system and the routine online death cause surveillance system in each DSP were first matched by an automatic computer checking algorithm. Persons included in both systems were identified as a match when national ID matched. If the national ID was missing, persons with the same name, gender and age (within three years) were used to identify a match. After an initial computer matching process, all mismatched cases were checked and verified by a further manual checking in the DSP level. The local staff checked each mismatched case with the records from the surveillance system. Missed death cases were identified after this thorough manual verification.

### Statistical methods

To test the conformity between under-reporting field survey data and the dataset of DSP system, we used a test of goodness of fit to calculate and compare the frequency distribution of main variables (age, cause of death, highest level of hospital where illness was diagnosed, and diagnostic basis) of the two datasets. The highest level of hospital where disease was diagnosed and the diagnostic basis were important indicators for accuracy of the underlying cause of death. Hospitals at the township-level and above were generally regarded as qualified to make correct diagnosis and the diagnoses made at village hospitals were checked and verified by senior DSP staffs. The diagnosis was considered reliable if it was made based on symptoms/signs, physio-biochemistry, pathology, autopsy or surgery. Inference-based diagnosis were verified with the original investigation documents.

We described the detailed steps of PS and CMR method as follows:

#### Propensity score weighting method

We used a propensity score weighting method based on a logistic regression of under-report, where the variables were selected stepwise. The inclusion criteria and the exclusion criteria were 0.1 and 0.12 respectively. The variables used for analysis included age, gender, rural/urban residency, geographic locations, educational attainment, occupation, marital status, cause of death, place of death and diagnostic unit. Geographic locations were classified as east, central and west according to criteria of National Bureau of Statistics. The cause of death was identified according to the International Statistical Classification of Diseases and Related Health Problems 10th Revision (ICD-10).

We used two groups (those aged 5 years and below and those above 5 years) to set up two separate models. The model included age, geographic location, urban and rural for children aged 5 years and below. Whereas for those over 5 years old, the model included age, gender, geographic location, occupation, rural/urban residency, marital status, place of death, diagnostic unit, cause of death and year of death. Propensity score weighting integrated the information of several major covariates into one propensity score variable. The estimated propensity score weighting may lead to a substantial reduction in bias, especially for small groups. The analytical procedure is as follows:

Step 1*: Model estimation*

The sampled under-reporting survey may not be perfectly representative of the whole DSP in terms of socioeconomic variables that are related to the probability a death is included in DSP. We applied logistic regression to the sociodemographic variables to predict the probability a respondent was included in the routine surveillance in the sampled under-reporting survey site, using all individual records in the under-reporting field survey of 2009–2011 as the gold standard. We used age, sex, place of death and other predictor variables in the model. The coefficient and standard error for each variable of the models are shown in Table 1 (for under 5 years) and Table 2 (for above 5 years). The goodness of fit reached 0.208 and 0.214 respectively. The regression equations for the two models were:

**Table 1:** Coefficient and standard error of variables in model 1 (for under 5 years)

Variable	Estimated coefficient	Standard error	z	p
Intercept	−1.28	0.12	122.42	<0.01
Urbanity(x_1_)(ref: rural)				
Urban	−0.187	0.097	3.75	0.053
Age(*x*_2_)(ref:0-1year)				
1-5 year	−0.249	0.076	10.67	<0.01
Year(x_3_) (ref: 2009)				
Year 2010	0.154	0.103	2.24	0.134
Year 2011	−0.011	0.11	0.010	0.922
Highest level of hospital where disease was diagnosed(x_4_) (ref: provincial level)				
City level	−0.432	0.18	5.49	0.02
County level	0.156	0.151	1.08	0.30
Township level	0.101	0.205	0.242	0.623
Village level	0.351	0.301	1.357	0.244
Other	−0.462	0.244	3.595	0.058
No treatment	0.134	0.408	0.109	0.742

**Table 2:** Coefficient and standard error of variables in model 2 (for above 5 years)

Variable	Estimated coefficient	Standard error	z	p
Intercept	−1.860	0.068	759.88	<0.01
Region(x_1_) (ref: East)				
Central	−0.183	0.015	147.83	<0.01
West	0.419	0.015	826.06	<0.01
Age(*x*_2_) (ref: 6–14 years)				
15-44years	−0.036	0.041	0.797	0.397
45-64years	−0.021	0.038	0.294	0.587
65 years or older	−0.070	0.038	3.485	0.062
Year(x_3_) (ref: 2009)				
Year 2010	−0.007	0.015	0.227	0.634
Year 2011	−0.068	0.015	21.856	<0.01
Highest level of hospital where disease was diagnosed (x_4_)(ref: provincial level)				
City level	−0.172	0.030	34.18	<0.01
County level	0.006	0.027	0.041	0.838
Township level	0.058	0.032	3.369	0.067
Village level	−0.152	0.046	10.894	0.001
Other	−0.178	0.051	12.030	<0.01
No treatment	0.582	0.090	41.720	<0.01
Marital status(x_5_) (ref: Married)				
Unmarried	0.095	0.054	3.093	0.079
Divorce	−0.391	0.100	15.357	<0.01
Widowed	−0.122	0.044	7.519	0.006
Unknown	0.428	0.123	12.178	<0.001
Education (x_6_) (ref: Illiteracy)				
Primary school	−0.015	0.031	0.240	0.624
Middle school	0.065	0.033	3.797	0.051
University or above	−0.022	0.073	0.089	0.766
Unknown	−0.076	0.079	0.931	0.335
Occupation(x_7_) (ref: peasant)				
Official and administrator	−0.017	0.053	0.105	0.746
Technical staff	−0.653	0.153	18.350	<.001
Clerk	−0.016	0.130	0.015	0.904
Self-employed	0.657	0.155	17.875	<.001
Worker	0.017	0.112	0.024	0.877
Unemployed and retired	−0.209	0.087	5.769	0.016
Other	0.379	0.047	65.934	<.001
Place of death(x_8_) (ref: Home)				
Hospital	−0.151	0.044	11.892	<0.001
On the way to the hospital	−0.190	0.070	7.351	<0.001
Other place	0.228	0.053	18.306	<0.001
Unknown	0.039	0.124	0.101	0.751
Cause of death(x_9_) (ref: other disease)				
Cancer	0.056	0.046	1.476	0.224
Cardiovascular disease	0.098	0.042	5.44	0.020
Respiratory disease	0.120	0.050	5.87	0.015
Nervous system disease	−0.137	0.09	2.23	0.135
Digestive system disease	−0.118	0.07	2.53	0.112
Urinary system disease	0.036	0.091	0.16	0.694
Congenital anomalies	−0.083	0.211	0.16	0.693
Injury	−0.056	0.053	1.12	0.291
Infectious disease	−0.153	0.067	5.14	0.023
Pregnancy, childbirth and the puerperium	0.057	0.265	0.047	0.829

Equation for model 1 (under 5 years):

$$ \begin{array}{l}\mathrm{Logit}\left(\mathrm{p}\right)=\hbox{-} 1.28\hbox{-} 0187{\mathrm{x}}_{1,2\_1}\hbox{-} 0.249{\mathrm{x}}_{2,2\_1}+0.15{\mathrm{x}}_{3,2\_1}\hbox{-} 0.011{\mathrm{x}}_{3,3\_1}\hbox{-} 0.432{\mathrm{x}}_{4,2\_1}+0.156{\mathrm{x}}_{4,3\_1}\\ {}+0.101{\mathrm{x}}_{4,4\_1}+0.351{\mathrm{x}}_{4,5\_1}\hbox{-} 0.462{\mathrm{x}}_{4,6\_1}+0.134{\mathrm{x}}_{4,7\_1}\end{array} $$

where x1 refers to urbanity, *x*2 refers to age group, x3 refers to year and x4 refers to the highest level of hospital where disease was diagnosed listed in Table 1.

Equation for model 2 (above 5 years):

$$ \begin{array}{l}\mathrm{Logit}\left(\mathrm{p}\right)=\hbox{-} 1.86\hbox{-} 0.183{\mathrm{x}}_{1,2\_1}+0.419{\mathrm{x}}_{1,3\_1}\hbox{-} 0.036{\mathrm{x}}_{2,2\_1}\hbox{-} 0.021{\mathrm{x}}_{2,3\_1}\hbox{-} 0.070{\mathrm{x}}_{2,4\_1}\hbox{-} 0.007{\mathrm{x}}_{3,2\_1}\\ {}\kern0.5em \hbox{-} 0.068{\mathrm{x}}_{3,3\_1}\hbox{-} 0.172{\mathrm{x}}_{4,2\_1}+0.006{\mathrm{x}}_{4,3\_1}+0.058{\mathrm{x}}_{4,4\_1}\hbox{-} 0.154{\mathrm{x}}_{4,5\_1}\hbox{-} 0.178{\mathrm{x}}_{4,6\_1}+0.582{\mathrm{x}}_{4,7\_1}\\ {}+0.095{\mathrm{x}}_{5,2\_1}\hbox{-} 0.391{\mathrm{x}}_{5,3\_1}\hbox{-} 0.122{\mathrm{x}}_{5,4\_1}+0.428{\mathrm{x}}_{5,5\_1}\hbox{-} 0.015{\mathrm{x}}_{6,2\_1}+0.065{\mathrm{x}}_{6,3\_1}\hbox{-} 0.022{\mathrm{x}}_{6,4\_1}\hbox{-} 0.\\ {}\kern0.1em 076{\mathrm{x}}_{6,5\_1}\hbox{-} 0.017{\mathrm{x}}_{7,2\_1}\hbox{-} 0.653{\mathrm{x}}_{7,3\_1}\hbox{-} 0.016{\mathrm{x}}_{7,4\_1}+0.657{\mathrm{x}}_{7,5\_1}+0.017{\mathrm{x}}_{7,6\_1}\hbox{-} 0.209{\mathrm{x}}_{7,7\_1}\\ {}+0.379{\mathrm{x}}_{7,8\_1}\hbox{-} 0.151{\mathrm{x}}_{8,2\_1}\hbox{-} 0.190{\mathrm{x}}_{8,3\_1}+0.228{\mathrm{x}}_{8,4\_1}+0.039{\mathrm{x}}_{8,5\_1}+0.056{\mathrm{x}}_{9,2\_1}+0.098{\mathrm{x}}_{9,3\_1}+\\ {}\kern0.1em 0.120{\mathrm{x}}_{9,4\_1}\hbox{-} 0.137{\mathrm{x}}_{9,5\_1}\hbox{-} 0.118{\mathrm{x}}_{9,6\_1}+0.036{\mathrm{x}}_{9,7\_1}\hbox{-} 0.083{\mathrm{x}}_{9,8\_1}\hbox{-} 0.056{\mathrm{x}}_{9,9\_1}\hbox{-} 0.153{\mathrm{x}}_{9,10\_1}+0.00\\ {}57{\mathrm{x}}_{9,11\_1}\end{array} $$

where x1 refers to region, *x*2 refers to age group, x3 refers to year, x4 refers to the highest level of hospital where disease was diagnosed, x5 refers to marital status, x6 refers to education, x7 refers to occupation, x8 refers to place of death and x9 refers to cause of death listed in Table 2.

Step 2*: Weighted estimates for death cases*

The probability of being reported for each observation (p_i_) was based on the logistic regression model of the field survey data. Weights for each case were calculated as w_i_ = 1/p_i_. The weighted number of deaths from 2009 to 2011 (Ts) was:

$$ {\mathrm{T}}_{\mathrm{s}}={\displaystyle \sum_{\mathrm{i}=1}^{\mathrm{Ns}}{\mathrm{W}}_{\mathrm{i}}} $$

Where N_s_ is the total number of death cases from the DSP 2009–2011 surveillance.

Theoretically, the sum of w_i_ of the cases represented the actual number of deaths, which was the total number of deaths that occurred during 2009–2011.

Step 3: The under-reporting rate of DSP from 2009–2011 (P) based on propensity score weighting was:

$$ \mathrm{P}=\left({\mathrm{T}}_{\mathrm{s}}-{\mathrm{N}}_{\mathrm{s}}\right)\ast 100\%/{\mathrm{T}}_{\mathrm{s}} $$

#### CMR method

To compare the results calculated from propensity score weighting method, we also used the CMR method to calculate the under-reporting rate. CMR has been widely used in wildlife science to estimate the size of free-living animal population and it has been advocated for use in estimating completeness of a registration [8]. In the two-sample capture-mark-recapture approach, an estimate of the true population size is derived assuming independence of ascertainment by evaluating the degree of overlap from existing data sources.

To perform CMR analysis, the estimated overall death toll (N) was

$$ \mathrm{N}=\left[\left(\mathrm{M}+1\right)\left(\mathrm{n}+1\right)/\left(\mathrm{m}+1\right)\right]-1 $$

where M is defined as the total number of cases in the routine DSP surveillance, n is defined as the total number of cases in under-reporting field survey, and m is defined as the number of cases reported in both systems.

The under-reporting rate of DSP from 2009–2011 (p) based on CMR was:

$$ p=\left(\mathrm{N}\hbox{-} \mathrm{M}\right)\ast 100\%/\mathrm{N} $$

## Results

### Baseline characteristics of database

Table 3 shows the comparison of the sample dataset and the DSP dataset. Less than 10 % of the death cases were diagnosed below township-level hospitals and more than 90 % were diagnosed with solid basis, implying the accuracy and good quality of cause of death reported by the DSP system. The comparison showed that there were no significant differences between the two sources in terms of the major variables. As shown in Table 4, the crude under-reporting rate from field survey was 12.6 % during the period 2009–2011. The crude rate decreased from 13.5 % in 2009 to 11.8 % in 2011 and higher in rural (14.1 %) compared to urban (10.7 %) areas. Among all age groups, the crude under-reporting rate was highest in the 0–5 year group (19.6 %) and lowest in the 65 years and above group (12.2 %).

**Table 3:** Test of goodness for fit of under-reporting field survey data and DSP dataset

Variable	Proportions in DSP dataset (Pi)	Proportions in under-reporting survey dataset (Si)	(Si-Pi)^2/Pi	χ^2^	*P*
Age					
0-5	0.6	1.3	0.817	0.312	>0.05
6-14	0.8	0.5	0.113		
15-44	8.1	8.0	0.001		
45-64	22.5	22.8	0.004		
65+	67.3	66.9	0.003		
Cause of death					
Cancer	23.8	24.1	0.004	1.239	>0.05
Cardiovascular disease	43.9	42.4	0.051		
Respiratory disease	9.0	11.1	0.490		
Nervous system disease	1.4	1.3	0.007		
Digestive system disease	2.4	2.3	0.004		
Urinary system disease	1.3	1.2	0.008		
Congenital anomalies	0.4	0.4	0.000		
Injury	8.2	9.0	0.078		
Infectious diseases	3.2	3.5	0.028		
Pregnancy, childbirth and the puerperium	0.5	0.6	0.020		
Other disease	5.9	4.1	0.549		
Highest level of hospital where disease was diagnosed					
Provincial level	14.2	12.6	0.180	1.876	>0.05
City level	28.0	25.8	0.173		
County level	34.2	40. 5	1.124		
Township level	13.1	12.3	0.049		
Village level	3.9	3.8	0.003		
Other	5.7	4. 5	0.257		
No treatment	1.0	0.7	0.090		
Diagnostic criteria					
Symptoms + physio-biochemistry	55.8	57.6	0.058	0.360	>0.05
Pathology	7.9	6.9	0.127		
Symptoms/signs	25.1	25.6	0.010		
Autopsy	0.6	0.6	0.000		
Surgery	1.7	1.4	0.053		
Inference	7.8	6.9	0.104		
Other	1.1	1.0	0.009		

^2=square

**Table 4:** Crude under-reporting rate of mortality from field survey 2009-2011^a^

Variable	Crude under-reporting rates			Total
	2009	2010	2011	
Geographic region				
East	10.2(1232/12026)	10.6(1325/12466)	8.9(1083/12173)	9.9
Central	12.1(1223/10114)	10.3(1044/10120)	10.6(1079/10211)	11.0
West	19.8(1630/8241)	18.0(1481/8229)	17.6(1496/8517)	18.4
Sex				
Male	13.1(2289/17470)	12.3(2173/17711)	11.8(2111/17900)	12.4
Female	13.9(1796/12911)	12.8(1677/13104)	11.9(1547/13001)	12.9
Rural/urban				
Urban	12.1(1584/13074)	11.1(1496/13473)	8.9(1196/13460)	10.7
Rural	14.5(2501/17307)	13.6(2354/17342)	14.1(2462/17441)	14.1
Age (years)				
0-5	17.4(76/438)	22.0(89/405)	19.6(64/326)	19.6
6-14	21.2(54/255)	17.8(48/270)	18.2(45/248)	19.0
15-44	13.9(372/2669)	14.4(345/2394)	13.8(327/2363)	14.1
45-64	14.1(960/6833)	12.1(827/6836)	11.6(818/7055)	12.6
65+	13.0(2623/20186)	12.2(2541/20910)	11.5(2404/20909)	12.2
Cause of death				
Cancer	11.8(849/7180)	10.2(747/7340)	10.1(746/7420)	10.7
Cardiovascular diseases	13.6(1784/13091)	13.2(1802/13683)	12.0(1641/13658)	12.9
Respiratory diseases	16.3(459/2810)	12.5(351/2802)	13.7(373/2715)	14.2
Nervous system diseases	9.4(39/413)	11.2(48/427)	11.4(49/431)	10.7
Digestive system diseases	12.4(93/749)	10.6(75/709)	10.5(78/740)	11.2
Urinary system diseases	11.7(47/403)	13.0(52/400)	11.6(43/371)	12.1
Congenital anomalies	17.9(22/123)	16.0(21/131)	17.2(20/116)	17.0
Injury	14.1(367/2610)	14.4(353/2448)	12.6(318/2516)	13.7
Infectious diseases	12.1(124/1029)	11.0(105/953)	11.1(111/997)	11.4
Pregnancy, childbirth and the puerperium	21.3(34/160)	27.3(39/143)	19.8(23/116)	22.9
Other diseases	14.7(267/1813)	14.5(257/1779)	14.1(256/1821)	14.4
Total	13.5(4085/30381)	12.5(3850/30815)	11.8(3658/30901)	12.6

^a^Data shown as rates (No. of under-reported cases/No. of total death cases)

### Under-reporting rate based on propensity score weighting and CMR

As shown in Table 5, using propensity score weighting method, the overall rate of under-reporting in the DSPs was 12.9 % (95%CI 11.2 %, 14.6 %) after weighting. The under-reporting rate was 12.7 % (11.0 %, 14.6 %), 13.1 % (11.3 %, 14.8 %) and 13.0 % (11.2 %, 14.6 %) in 2009, 2010 and 2011 respectively. The under-reporting rate gradually decreased for deaths at higher ages. The rate was highest in the age group 0–5 years (23.7 %, 95%CI 16.1 %, 35.5 %) and lowest in the age group over 65 years (12.4 %, 95%CI 10.9 %, 13.6 %). The under-reporting rate was higher in the west (18.8 %, 95%CI 16.5 %, 21.0 %) than the east (10.1 %, 95%CI 8.6 %, 11.3 % and central regions (11.2 %, 95%CI 9.6 %, 12.7 %).

**Table 5:** Under-reporting rates and 95%CI based on CMR and propensity score weighting^a^

	Under-reporting rate based on CMR(%)				Under-reporting rate based on propensity score weighting (%)			
	2009	2010	2011	Total	2009	2010	2011	Total
Geographic region								
East	10.2(9.7,10.8)	10.6(10.1,11.2)	8.9(8.4,9.4)	9.9(9.6,10.2)	9.9(8.4,11.0)	10.1 (8. 7,11.3)	10.2 (8. 7,11.3)	10.1(8.6,11.3)
Central	12.1(11.5,12.7)	10.3(9.7,10.9)	10.6(10.0,11.1)	11.0(10.7,11.3)	11.1(9.5,12. 6)	11.4 (9.7,13.0)	11.1(9.7,13.0)	11.2(9.6,12. 7)
West	19.8(18.9,20.1)	18.0(17.2,18.8)	17.6(16.8,18.3)	18.4(18.0,18.9)	18.5(16.2,20.7)	19.0 (16.7,21.3)	18.8 (16.7,21.3)	18.8(16.5,21.0)
Sex								
Male	13.1(12.6,13.6)	12.2(11.2,13.2)	11.8(11.3,12.2)	12.4(12.3,12.8)	12.5 (11.3,14.5)	13.0(11.1,14.6)	12.8 (11.0,14. 5)	12.8(11.0,14.4)
Female	13.9(13.3,14.5)	12.8(12.2,13.4)	11.9(11.4,12.4)	12.9(12.5,13.2)	13.0(10.8,14.2)	13.3 (11. 6,14.9)	13.2 (11.5,14.8)	13.2(11.4,14.7)
Rural/urban								
Urban	12.1(11.6,12.7)	11.1(11.0,11.6)	8.9(8.4,9.4)	10.7(10.4,11.0)	11.0 (9.3,12. 6)	11.4(9.6,13.0)	11.3(9.6,12.8)	11.2 (9.5,12. 8)
Rural	14.5(13.9,15.0)	13.6(13.1,14.1)	14.1(13.6,14.6)	14.1(13.8,14.3)	13.6 (11.9,15.3)	14.0 (12.2,15.7)	13.9 (12.1,15.6)	13.9(12.1,15.5)
Age(years)								
0-5	17.3(13.7,20.6)	21.9(17.8,25.7)	19.6(15.1,23.6)	19.6(17.3,21.7)	24.0(16.3,36.0)	23.6(16.0,35.4)	23.6 (16.1,35.1)	23.7 (16.1,35.5)
6-14	21.1(16.0,25.6)	17.7(13.2,21.8)	18.1(13.3,22.4)	19.0(16.3,21.6)	16.0(12.7,19.4)	15.7 (12.5,19.1)	16.4 (13.1,20.0)	16.0 (12.7,19.5)
15-44	13.9(12.6,15.2)	14.4(13.0,15.8)	13.8(12.5,15.2)	14.1(13.3,14.8)	14.2(12.0,16.3)	14.8 (12.5,17.0)	14.8(12. 5,16.9)	14.6(12.3,16.7)
45-64	14.1(13.2,14.8)	12.1(11.3,12.8)	11.6(10.9,12.3)	12.6(12.1,13.0)	12.5 (10.9,13.8)	13.0 (11.3,14.4)	12.8 (11.2,14.2)	12.8(11.1,14.2)
65+	13.0(12.5,13.4)	12.2(11.7,12.6)	11.5(11.1,11.9)	12.2 (12.0,12. 5)	12.2 (10.9,13.8)	12.6 (11.0,13.8)	12.5(10.9,13.7)	12.4 (10.9,13.6)
Cause of death								
Cancer	11.8(11.1,12.5)	10.2(9.5,10.8)	10.1(9.4,10.7)	10. 7(10.3,11.1)	10.5 (9.1,11. 6)	11.0(10.0,16.4)	10.9(9.4,12.0)	10.8(9.4,11.9)
Cardiovascular disease	13.6(13.1,14.2)	13.2(12.6,13.7)	12.0(11.5,12.5)	12.9 (12.6,13.2)	13.0(11.5,14.1)	13.3 (11.8,14.6)	13.2(11.7,14.4)	13.2(11.6,14.3)
Respiratory disease	16.3(15.0,17.6)	12.5(11.3,13.7)	13.7(12.5,15.0)	14.2 (13.5,14.9)	14.1 (12.5,15.4)	14.7 (13.0,16.1)	14.7(13.0,16.0)	14.5 (12.8,15.8)
Nervous system disease	9.4(6.6,12.1)	11.2(8.2,14.0)	11.3(8.4,14.1)	10.7(9.0,12.3)	11.1 (8.8,13.5)	11.4 (9.0,13.8)	11.2 (8.9,13. 6)	11.3(8.9,13.6)
Digestive system disease	12.4(10.1,14.6)	10.6(8.3,12.7)	10.5(8.3,12.6)	11.2(9. 9,12.4)	11.3(9.2,13.3)	11.7(9.5,13.8)	11.6 (9.5,13.8)	11.5 (9.4,13.6)
Urinary system disease	11.6(8.5,14.6)	13.0(9.7,16.0)	11.6(8.3,14.6)	12.1(10.2,13.9)	11.6 (9.3,13.9)	12.2(9.8,14.5)	11.9 (9.6,14.2)	11.9 (9.6,14.2)
Congenital anomalies	17.8(10.7,23.8)	15.9(9.4,21.5)	17.1(9.9,23.2)	17.0(13.1,20.5)	20.3 (13.7,30.2)	20.3 (13.6,30.4)	20.6(14.1,29.4)	20.4(13.8,30.0)
Injury	14.1(12.7,15.9)	14.4(13.0,15.8)	12.6(11.4,13.9)	13.7 (12.9,14. 5)	14.1 (11.6,17.2)	14.7 (12.2,17.6)	14.6(12.0,17.5)	14. 5(11.9,17.4)
Infectious diseases	12.0(10.1,13.9)	11.0(9.0,12.9)	11.1(9.2,13.0)	11.4 (10.3,12.5)	11.9 (9.5,14.6)	12.1(9.6,15.0)	12.0(9.6,14. 6)	12.0(9. 6,14.7)
Pregnancy, childbirth and puerperium	21.1(14.5,26.8)	27.1(19.3,33.5)	19.7(12.0,26.1)	22.9(18.7,26.6)	25.7 (18.7,34.8)	26.1 (18.8,36.0)	26.1 (18. 8,36.0)	26.0(18.8,35.6)
Other diseases	14.7(13.1,16.3)	14.4(12.9,16.0)	14.1(12.5,15.6)	14.4 (13.5,15.3)	14.9(12.5,17.3)	14.2 (12.0,16.4)	14.4 (12.2,16.5)	14.5 (12.3,16.7)
Total	13.5(13.1,13.8)	12.5(12.1,12.9)	11.8(11.5,12.2)	12.6(12.4,12.8)	12.7 (11.0,14.6)	13.1 (11.3,14.8)	13.0(11.2,14.6)	12.9 (11.2,14.6)

^a^data shown as under-reporting rate (95%CI). CI: Confidence Intervals

According to the CMR method, the overall under-reporting rate of DSPs was 12.6 % (12.4 %, 12.8 %). The under-reporting rate was 10.7 % (10.4 %, 11.0 %) in urban and 14.1 % (13.8 %, 14.3 %) in rural areas respectively. Consistent with the propensity weighting method, the under-reporting rate in the west was higher than the east and central regions (18.4 %, 9.9 % and 11.0 % respectively). The under-reporting rate for children aged 5 and below (19.6 %, 95%CI 17.3 %, 21.7 %) was the highest among all age groups.

### Life tables

Table 6 summarizes the outputs of unadjusted and adjusted life tables for males and females in the DSP. The death probability for the 0–5 year age group was 0.0118 and 0.0082 for males and females respectively. Life expectancy at birth is a comprehensive reflection of mortality among all age groups and this study showed that life expectancy for Chinese males and females was 77.3 and 86.4 before adjustment. The under-reporting-adjusted life expectancy was 75. 7 and 81.9 for males and females respectively.

**Table 6:** Summary outputs of unadjusted and adjusted life tables based on the propensity score weighting method

	Male		Female	
	Unadjusted	Adjusted	Unadjusted	Adjusted
Life expectancy at birth	77.3	75. 7	86.4	81.9
Risk of dying between 0–5 years	0.0098	0.0118	0.0070	0.0082
Risk of dying between 15–60 years	0.1188	0.1307	0.0564	0.0615
Life expectancy at age 60 years	22.4	21.2	29.6	24.9

## Discussion

The adjusted under-reporting rate for mortality during 2009–2011 using both methods in our study decreased compared to the period 2006–2008. Consistent with previous studies, we found a significantly higher under-reporting rate in rural areas than in urban areas [7]. This could largely be explained by lack of experienced doctors in charge of completing the death report and inconvenience of information transfer. Additionally, the unwillingness of reporting in many bereaved families in rural areas worsened the under-reporting situation [9, 10]. Similarly, the higher under-reporting rate in the west compared with east and central regions was mainly caused by lack of personnel and technical resources in less developed areas. Moreover, the special customs of some ethnic minorities in western regions made them less likely to report the death cases, especially for infants. Not surprisingly, the under-reporting rate for the population aged 5 years and below was the highest among all age groups. This may be associated with the poor quality of the death report card for infants and young children. Stigma and shame may lead some parents to shelter the facts of children’s death, particularly in rural areas and western regions. Furthermore, in the floating population (a group of people who do not live in the area permanently and are not considered official residents) of urban migrants, health services for mothers and children under 5 years of age are more difficult to access [11].

In under-reporting surveys, populations often display dependence and heterogeneity. A model of a stable population can always be imposed if using CMR. It is difficult, however, to have independent samples and this would lead to inaccurate and sometimes misleading results [12]. It is not possible to evaluate the possible under-reporting rate when there are only two ascertainment sources, such as under-reporting survey and DSPs. Quality criteria about survey performance was defined for all populations in DSPs. The advantage of the CMR method to calculate the under-reporting rate is simplicity and ease of practical use. However, the CMR results could appear large deviation if covariance distribution between groups is uneven when using CMR method to calculate the under-reporting rate for subgroups. The propensity score weighting method is used to make observational data look like random distribution, and the results show that propensity score weighting estimates are more internally consistent than the cell based approach.

In a sampled field survey like the under-reporting survey, it is not easy to meet all the conditions.

Dependencies between the individual cases make it easy for some deaths to be captured in some groups as opposed to others. It is more likely to be captured by another source. When calculating the under-reporting rates for different groups, selection bias would lead to biased results. When the distribution of covariates is consistent as in the current study, the results of the two methods are similar. However, the propensity score weighting method is more flexible and suitable to calculate the under-reporting rates for different population groups because it takes into account each individual death.

The propensity score weighting method represents the influence of multiple covariates for under-reporting. It reduces the dimension of covariates and calculated under-reporting rate of each group based on the scores. In a large sample of cases, individuals between the groups could be adjusted using propensity score, making the distribution of covariates between the groups equivalent to achieve a post-randomization [13]. Furthermore, propensity score weighting estimates are internally consistent, especially for the group with fewer death cases. For example among the population of >5 years age group, fewer deaths in the 6–14 year age group led to a big selection bias, and the under-reporting rate in this group was much higher than other groups based on CMR method. Propensity score weighting eliminated the bias, so the under-reporting rate in the 6–14 year age group based on PS was closer to the average rate in the >5 years age group. The results of propensity score weighting were therefore closer to the true level of under-reporting.

The reasons for under-reporting are multifaceted, such as the local government's emphasis on the work, competence and responsibility on the staff, affection of the local death registration system and collaboration of government departments [7, 11, 14, 15]. Local population migrations and the traditional concept of folk culture are also possible reasons for under-reporting. The fundamental way to improve the quality of data is not through under-reporting rate adjustments, but by improving and strengthening the quality management system. All levels of government should increase investment in mortality surveillance, especially in rural areas and western regions. Communications and coordination with the local public security departments and other relevant departments and sectors need to be strengthened to allow multi-channel data complement. The enthusiasm of rural health centers or community health service doctors should be mobilized to report the death cards more carefully and accurately, and they should play a key role in the data collection process.

The calculation of life expectancy in a population relies on the accurate estimate of the age-specific death rate. Using propensity score weighting based under-reporting rates, the generated adjusted life tables for the DSP population will shed light on the implications of under-reporting for assessment of mortality patterns in China. The results for people aged above 5 years in the current study were similar to the estimation of Global Burden of Disease China Study [16]. The higher life expectancy at birth of our study is due to the relatively lower under-reporting rate for the under 5 year age group in DSP system. There was separate death surveillance for the under 5 year population in the Maternal and Child Health Surveillance Center of China (MCHSCN). The combination of the data from DSP and MCHSCN would produce the most accurate estimate of mortality in all age groups in China.

With the rapid economic development and urbanization in China, floating population has increased gradually and became an important part of the Chinese population. The current death cause surveillance system focuses on the residents who have lived in the DSP site for more than six months (considered as locally registered residents). Therefore we were not able to obtain death information for the population who had lived in the DSP site for less than six months. Mortality of this group is hard to track and they had potential impact on the overall mortality of Chinese population. The Chinese government has realized the importance of evaluating the health status of the floating population and initiated a national chronic disease risk factor survey based on the DSP system [17]. More investments are expected for this group and the floating population will be included in the death cause surveillance in future exercises.

There are some limitations of PS method. Firstly, the under-reporting was influenced by many sociodemographic variables of the death individuals. Since such information came from the death cards, incomplete and inaccurate records of the death individuals entered by the local staffs would affect the accuracy of logistic regression model. Secondly, although the PS method can eliminate some errors caused by sampling selection, it is not possible to get a perfectly random distribution. In addition, the PS method is more complicated and not easy for practical use compared to the CMR method. Furthermore, death information for the floating population is incomplete in the current death cause surveillance system and we were not able to estimate the true mortality of this group as an important component of the population in China.

The Chinese government has planned to expand the current death cause surveillance system to include more counties and districts with provincial representativeness. The propensity score weighting approach could be applied to estimate the under-reporting rates nationally and provincially to assess the quality of mortality data from the DSP system. The mortality data from DSP system need adjustment for under-reporting. Although both CMR and PS methods can do the adjustment, the latter utilizes much more information and should be more suitable to adjust DSP data. Overall, the results of propensity score weighting are more accurate and can be used to address under-reporting in mortality surveillance in China.
